# Operational Modal Analysis of Bridge Structures with Data from GNSS/Accelerometer Measurements

**DOI:** 10.3390/s17030436

**Published:** 2017-02-23

**Authors:** Chunbao Xiong, Huali Lu, Jinsong Zhu

**Affiliations:** 1 Key Laboratory of Coast Civil Structure Safety, Tianjin University, Ministry of Education, Tianjin 300072, China; luhai_tj@126.com; 2 School of Civil Engineering, Tianjin University, Tianjin 300072, China; huali_lu@tju.edu.cn

**Keywords:** operational modal analysis, GNSS dynamic deformation monitoring, accelerometer, AFEC mixed filtering, expanded ARMA_RDT

## Abstract

Real-time dynamic displacement and acceleration responses of the main span section of the Tianjin Fumin Bridge in China under ambient excitation were tested using a Global Navigation Satellite System (GNSS) dynamic deformation monitoring system and an acceleration sensor vibration test system. Considering the close relationship between the GNSS multipath errors and measurement environment in combination with the noise reduction characteristics of different filtering algorithms, the researchers proposed an AFEC mixed filtering algorithm, which is an combination of autocorrelation function-based empirical mode decomposition (EMD) and Chebyshev mixed filtering to extract the real vibration displacement of the bridge structure after system error correction and filtering de-noising of signals collected by the GNSS. The proposed AFEC mixed filtering algorithm had high accuracy (1 mm) of real displacement at the elevation direction. Next, the traditional random decrement technique (used mainly for stationary random processes) was expanded to non-stationary random processes. Combining the expanded random decrement technique (RDT) and autoregressive moving average model (ARMA), the modal frequency of the bridge structural system was extracted using an expanded ARMA_RDT modal identification method, which was compared with the power spectrum analysis results of the acceleration signal and finite element analysis results. Identification results demonstrated that the proposed algorithm is applicable to analyze the dynamic displacement monitoring data of real bridge structures under ambient excitation and could identify the first five orders of the inherent frequencies of the structural system accurately. The identification error of the inherent frequency was smaller than 6%, indicating the high identification accuracy of the proposed algorithm. Furthermore, the GNSS dynamic deformation monitoring method can be used to monitor dynamic displacement and identify the modal parameters of bridge structures. The GNSS can monitor the working state of bridges effectively and accurately. Research results can provide references to evaluate the bearing capacity, safety performance, and durability of bridge structures during operation.

## 1. Introduction

Various types of damage and functional degradation are inevitable due to the ageing of materials and the environmental erosion of bridges and increasing overload vehicles. Dynamic deformation monitoring and real-time state evaluation of bridges in service are important to protect the carrying capacity, durability, and safety of bridge structures; provide early warning against emergency; and prevent huge economic loss and casualties under extreme conditions [1].

Traditional monitoring methods of bridge structures requires sufficient test equipment, including accelerometers, strain gauges, and inclination sensors. This test equipment brings a heavy workload and suffers from immense environment-induced losses. Furthermore, this equipment can only measure the high-frequency dynamic displacement of the structure and cannot test low-frequency quasi-static displacement [2]. The GNSS receivers feature easy operation, high environmental adaptation, satellite signal reception at any time, and long-term continuous monitoring. Additionally, the GNSS easily identifies low-frequency signals of structural vibration and can offset the disadvantages of traditional monitoring methods. The GNSS is increasingly appreciated in large-scale structural health monitoring as all test equipment can be developed continuously when combined with traditional monitoring equipment, such as an accelerometer [3,4,5]. Roberts et al. [6] designed a GNSS-accelerometer combined device in which the GNSS receiver was overlaid with the vertical axis of the accelerometer. Li et al. [7] and Moschas et al. [8] monitored the vibration responses of iron tower and steel bridge structures by combining an accelerometer and a GNSS receiver. Yi et al. [9] presented an overview of research and development activities in the field of bridge health monitoring using the global positioning system (GPS).

Due to multiple errors, the GNSS has a high measurement accuracy to bridge with large vibration displacement and good flexibility. Nevertheless, with the continuing perfection and development of the GNSS hardware and software, the GNSS technology is being gradually used in the experimental study of bridge structures with relatively small amplitudes. Li et al. [10,11] designed an efficient multi-GNSS real-time precise positioning service system and presents a new GPS data processing scheme for real-time kinematic precise point positioning in order to shorten the convergence time and the observational time required for a reliable ambiguity-fixing. Li et al. [12] presented an approach for tightly combing GPS and seismic sensor data where the accelerometer data are integrated into the ambiguity-fixed PPP (precise point positioning) processing on the observation level. Lee et al. [13] proposed a detection, identification, and adaptive technique to improve the reliability of the GNSS positioning solution. Larocca et al. [14] monitored the dynamic features of small concrete bridge structures using two GPS receivers with a 100 Hz sampling frequency. Ogundipe et al. [15] performed GNSS monitoring of a viaduct with a steel box beam in Avonmouth, Bristol, England. They measured the vertical deflection at the main span (approximately 50 mm) point accurately and identified the natural frequency of vibration that conformed to finite element calculation results. Moschas et al. [16] implemented a dynamic response monitoring test to a steel footbridge with a 41.5 m main span range by using a GNSS receiver and an accelerometer. They measured the millimeter-scale dynamic displacement of a bridge structure and identified the vertical and horizontal frequencies of vibration. In this study, the real-time dynamic responses of the Tianjin Fumin Bridge in China were tested by combining the GNSS-Real Time Kinematic (RTK) technique and acceleration sensing. Real dynamic displacement of the bridge structure was extracted, and its vertical vibration performance was analyzed.

In the past, the GNSS-based measurement method for bridge monitoring was mainly used for real dynamic displacement extraction, which is infrequently used in modal parameter identification. Identification of modal parameters is important in the theoretical research and engineering applications of health monitoring, damage assessment, and the active control of bridge structures [17,18,19]. Given the increasing span and complexity of modern bridge structures, gaining effective excitation signals through human motivation (such as shakers and drop weights) is difficult, and may cause unnecessary damage to bridge structures [20]. Therefore, traditional theories and techniques concerning the identification of structural modal parameters based on input and output signals are not applicable. Identification methods of bridge structural modal parameters under ambient excitation (e.g., wind-induced vibration, earth pulse, and stimulus of vehicles) requires no human stimulus, only the mathematical and mechanical analyses of output signals as well as the identification of modal parameters in the time or frequency domains. These methods are feasible and practical in engineering applications. At present, the main modal parameter identification methods based on ambient excitation includes the random decrement technique (RDT) [21]; the natural excitation technique (NExT) [22]; the Ibrahim time domain technique (ITD) [23]; the ARMA [24]; and the random subspace method [25] in the time domain. In addition, some identification methods in the frequency domain can be used to identify modal parameters under ambient excitation; these methods include the peak-picking technique and the frequency-domain decomposition technique. In this study, the time-domain modal parameters of the preprocessed GNSS monitoring data were identified, and the traditional RDT for stationary random processes was expanded to non-stationary random processes. An improved identification method of ARMA_RDT modal parameters was applied to analyze the real-time dynamic monitoring data of the GNSS and extract the modal frequency of bridge structural systems in service.

To increase the measurement accuracy of the GNSS dynamic monitoring and expand it for the application of modal parameters identification, the dynamic responses of the Tianjin Fumin Bridge were monitored and tested by combining the GNSS sensor technology and acceleration sensing. An AFEC mixed filtering algorithm was proposed to extract the real vibration displacement of the bridge structure. Modal parameters of the bridge structure were identified using the expanded time-domain modal identification method (expanded ARMA_RDT) and then compared with the results of traditional acceleration sensor testing and finite element analysis. In this paper, Section 2 introduces the principle of the GNSS dynamic deformation monitoring. Section 3 presents the AFEC mixed filtering algorithm. Section 4 states the theory of the expanded ARMA_RDT modal identification method. Section 5 discusses engineering applications, and Section 6 presents our conclusions.

## 2. Principle of GNSS Dynamic Deformation Monitoring

**Figure 1:**
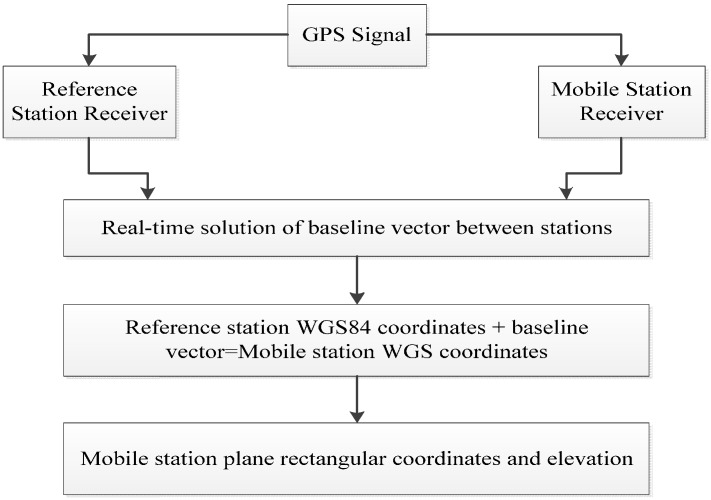
GNSS-RTK (Global Navigation Satellite System Real-time Kinematic Technology) working mode.

GNSS refers to all satellite navigation systems including American GPS; Russian Glonass; European Galileo; China’s Beidou navigation systems; and related enhancement systems. According to the characteristics of monitoring objects, GNSS deformation monitoring is divided into periodic measurement; fixed continuous GPS station array; and real-time dynamic monitoring. The first two types use static data calculation as the monitoring objects are generally viewed as static changes for slow deformation and long periods. In general, RTK monitoring is used to reduce the influences of different system errors and obtain real-time high-accuracy coordinates of measuring points. RTK monitoring is a real-time differential GPS technique based on the observed quantity of carrier phase, which is mainly applicable to long-term deformation with sudden changes and vibration deformation under ambient excitation. The working principle of the RTK technique is to set one GPS receiver at the reference station to observe all visible GPS satellites continuously and return real-time monitoring data to mobile stations. GPS receivers on mobile stations receive the observed data from the reference station through radio-receiving equipment while receiving GPS satellite signals. Subsequently, they perform real-time calculations and display 3D coordinates and accuracy of the user stations according to the principle of relative positioning [9]. The working mode is shown in Figure 1.

**Figure 2:**
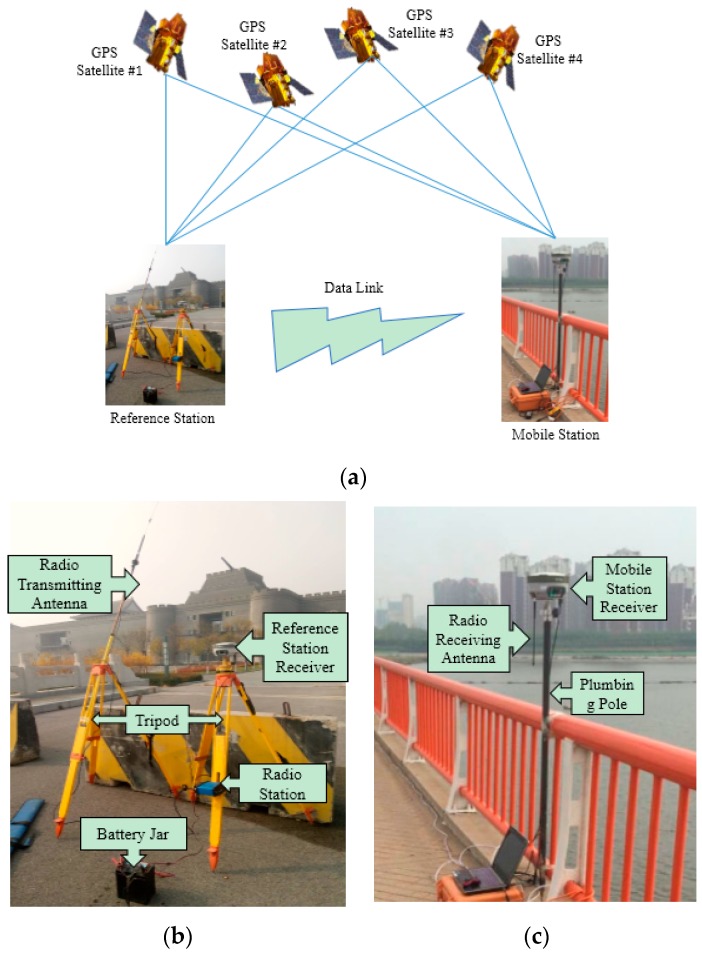
GNSS-RTK system composition where (**a**) is the GNSS-RTK system; (**b**) is the reference station; and (**c**) is the mobile station.

The GNSS-RTK system includes earth-circling satellites, a reference station, and mobile stations (Figure 2a). The reference station is composed of a GPS receiver, a satellite earth antenna, a wireless data link transceiver and transmitting antenna, and a direct-current power supply (Figure 2b). The mobile station is composed of a GPS receiver, a satellite earth antenna, a wireless data link receiver and antenna, and a data collector (Figure 2c).

## 3. AFEC Mixed Filtering

In general, the GNSS has a short baseline (<10 km) in structural dynamic monitoring, which can reduce errors caused by the troposphere and the ionized layer to some extent but cannot decrease multipath errors and random noise [26]. Therefore, the GNSS monitoring signals mainly cover real structural vibration information, multipath errors, and random noise. Multipath errors are mainly distributed in the 0–0.2 Hz frequency band [27], whereas random noise is distributed in a wide frequency band and has low energies. Given that the GNSS monitoring data contain countless noises and the vertical error is approximately 20 mm, the GNSS has difficulty in identifying millimeter-scale real vibrations of medium-sized and small bridge structures. An AFEC mixed filtering algorithm was proposed to solve these problems. Chebyshev filtering is applicable to ripple-like frequency response amplitudes on the pass-band or stop-band and is often used to filter structural vibration signals. The EMD technique decomposes signals from small to big according to the characteristic scale of local time, obtaining limited intrinsic modal functions (IMFs) from large to small frequencies. This technique has the advantages of multi-resolution and solution of the wavelet base selection in wavelet transform. Moreover, the EMD technique decomposes signals from scale characteristics of signals. It possesses good local adaptation and adaptability, and is convenient for filtering and de-noising non-stationary signals.

Signal *X_i_* can be gained by preprocessing signal *x_i_* collected by the GNSS and neglecting the minor constituent. In the dynamic deformation monitoring of the structure of GNSS, the measurement error is caused by many factors. In GNSS short-baseline double-difference solution processing, errors caused by tropospheric and ionospheric delays, satellite ephemeris errors, satellite clock errors, receiver clock errors and receiver position errors can be weakened. Some errors can’t be weakened, such as multi-path error and random noise. Ignoring minor components, such as the errors caused by earth tides and load tide, the preprocessed signal *X_i_* can be expressed as

(1) $$X_{i}=R_{i}(n)+D_{i}(n)+N_{i}(n)$$

where *X_i_* is the GNSS test data at the test point *i* after preprocessing; *n* is the data length; $R_{i}(n)$ is the real vibration information of the bridge structure; $D_{i}(n)$ is the multipath errors of signals; and $N_{i}(n)$ is the random noise of signals.

**Figure 3:**
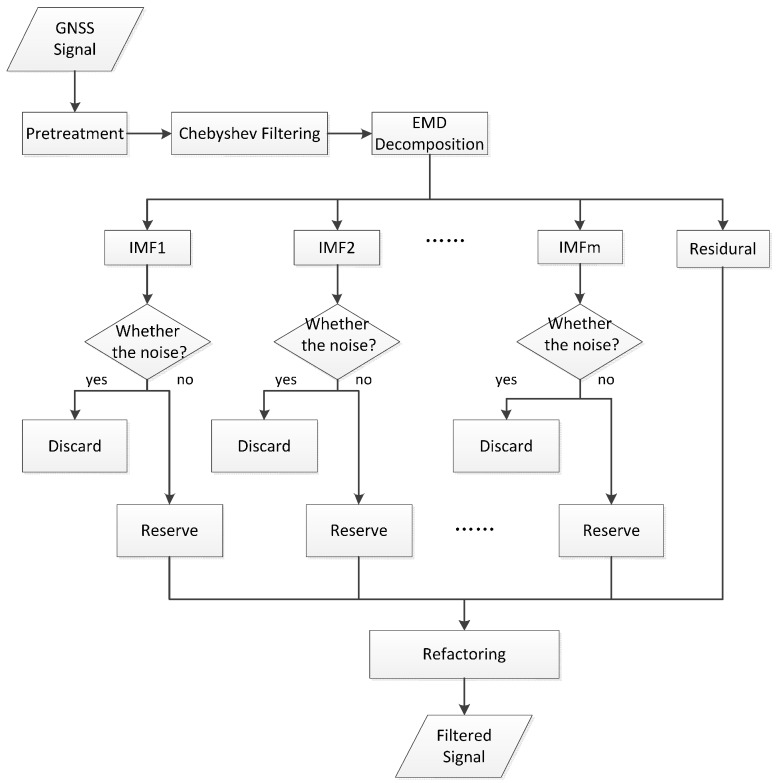
AFEC mixed filtering (an algorithm combing autocorrelation function-based empirical mode decomposition and Chebyshev filter) flow chart.

AFEC mixed filtering has two steps (Figure 3):

(1) Multipath errors $D_{i}(n)$ are eliminated by Chebyshev high-pass filtering, and the fundamental vibrational frequency of the test bridge structure was calculated as 0.83 Hz by using finite element analysis. The pass-band frequency of an 8-order I-type Chebyshev high-pass filter was designed at 0.4 Hz. This 8-order I-type Chebyshev high-pass filter was used to process the GNSS monitoring data *X_i_*, obtaining the signal after the multipath error was eliminated: $Y_{i}=N_{i}(n)+R_{i}(n)$.
(2) The random noise $N_{i}(n)$ was weakened by the EMD filter based on the autocorrelation function. First, the EMD of the signal *Y_i_* was implemented, obtaining *m* intrinsic modal functions (IMF1, IMF2,…, IMFm) and one residual component (*R*_1_). The autocorrelation function of *m* IMFs was calculated, and whether these IMFs were noise components was determined. The IMFs that were not noise components and the residual component were reconstructed, obtaining the AFEC-filtered signal $y_{i}=R_{i}(n)$.

## 4. Expanded ARMA_RDT

The program of the time-domain identification technique of modal parameters under ambient excitation included the following steps: (1) the collection of vibration data under ambient excitation; (2) the preprocessing of data samples to make them conform to the signal form necessary for time-domain identification. In this paper, expanded RDT was used to extract free vibration signal data from vibration response signals; (3) Identification of parameters by using free vibration signals as the input data of the time series method of the ARMA model. One advantage of the time-domain identification of modal parameters was that it only uses measured response signals and does not need Fourier transform, thus avoiding truncation-induced signal missing and influences on identification accuracy by side lobe and low resolution.

### 4.1. Expanded RDT

RDT is a method that extracts free attenuation vibration signals of the system through average and mathematical statistical methods. The traditional RDT focuses mainly on stationary random processes. For a single-degree-of-freedom structural system under excitation by zero-mean stationary Gaussian signals, the traditional RDT chooses one threshold A and intercepts the response signal of the system under random excitations, obtaining signals of *n* sections of time sequences starting at *t_i_* (*i* = 1, 2, …, *n*) and having a length of *s*. Subsequently, the signals of these *n* sections are superposed and averaged, thus obtaining the free vibration signal of the system. According to theoretical deduction, this vibration signal is the free attenuation signal of the structural system under initial displacement [21]. When the random excitation meets zero-mean Gaussian distribution, theoretical deduction reveals that this vibration signal is the free attenuation signal of the structural system under initial displacement.

(2) $$\delta (s)=\frac{1}{n}\sum_{i=1}^{n}x(t_{i}+s)$$

However, external excitations to bridge structures in service are mainly non-stationary random loads. This requires the expansion of the traditional RDT to process zero-mean non-stationary environmental response signals, thereby extracting the free attenuation signal of the multi-degree-of-freedom (MDOF) linear bridge structural system. Under zero-mean non-stationary random loads, the free vibration signal of the MDOF system is collected by RDT. The dynamic differential equation of the MDOF construction structural system can be expressed as:

(3) $$M\ddot{x}(t)+C\dot{x}(t)+Kx(t)=f(t)$$

where M, C and K are the total mass, damping, and stiffness matrixes of the structure, respectively; x(t), $\dot{x}(t)$ and $\ddot{x}(t)$ are the displacement, velocity, and acceleration vectors, respectively; and f(t) is the zero-mean non-stationary external load vector.

According to the basic principle of structural dynamics, the displacement response of the *i*th DOF in the linear structural system can be expressed as the superposition of multiple modal displacement:

(4) $$y_{i}(t)=\sum_{r=1}^{m}\varphi_{ir}\cdot q_{r}(t)$$

where $\varphi_{ir}$ is the *r*-order mode of vibration; $q_{r}(t)$ is the displacement response of the *r*-order modal; and *m* is number of order of the modal. $q_{r}(t)$ can be expressed as the sum of additional free vibration $q_{r1}(t)$ and forced vibration $q_{r2}(t)$ of the modal:

(5) $$\begin{array}{l} q_{r}(t)=q_{r1}(t)+q_{r2}(t)=e^{-\xi_{r}\omega_{r}t}[q_{r}(0)\cos\omega_{dr}t+\frac{{\dot{q}}_{r}(0)+\xi_{r}\omega_{r}q_{r}(0)}{\omega_{dr}}\sin\omega_{dr}t]\\ \;\;\;\;\;\;\;\;\;\;\;\;+\int_{0}^{t}\varphi_{r}^{T}f(\tau )h_{r}(t-\tau )d\tau \end{array}$$

where $\omega_{r}$ and $\xi_{r}$ are the inherent frequency and damping ratio of the *r*-order modal of the structural system, respectively; $\omega_{dr}$ is the damped frequency of the *r*-order modal; $h_{r}(t-\tau )$ is the unit impulse response function of the *r*-order modal; and $q_{r}(0)$ and ${\dot{q}}_{r}(0)$ are the initial modal displacement and initial modal velocity of the *r*-order modal, respectively.

Given that the unit impulse response will attenuate, $h_{r}(t-\tau )$ has a finite length. Considering the unilateral properties of the modal load and modal impulse response function, the researchers obtain:

(6) $$\int_{0}^{t}\varphi_{r}^{T}f(\tau )h_{r}(t-\tau )d\tau =\int_{0}^{p}\varphi_{r}^{T}h_{r}(\tau )f(t-\tau )d\tau$$

where *p* is the length of the modal impulse response function. Replacing *t* by *t_i_* + *s* yields:

(7) $$\begin{array}{l} q_{r}(t_{i}+s)=e^{-\xi_{r}\omega_{r}(t_{i}+s)}[q_{r}(0)\cos\omega_{dr}(t_{i}+s)+\frac{{\dot{q}}_{r}(0)+\xi_{r}\omega_{r}q_{r}(0)}{\omega_{dr}}\cdot \\ \;\;\;\sin\omega_{dr}(t_{i}+s)]\;+\int_{0}^{p}\varphi_{r}^{T}h_{r}(\tau )f(t_{i}+s-\tau )d\tau \\ \;\;\;=e^{-\xi_{r}\omega_{r}s}(A\cos\omega_{dr}s+B\sin\omega_{dr}s)\;\;+\int_{0}^{p}\varphi_{r}^{T}h_{r}(\tau )f(t_{i}+s-\tau )d\tau \end{array}$$

where *A* and *B* are:

(8) $$\begin{array}{l} A=e^{-\xi_{r}\omega_{r}t_{i}}\left(q_{r}(0)\cos\omega_{dr}t_{i}+\frac{{\dot{q}}_{r}(0)+\xi_{r}\omega_{r}q_{r}(0)}{\omega_{dr}}\sin\omega_{dr}t_{i}\right)\\ B=e^{-\xi_{r}\omega_{r}t_{i}}\left(q_{r}(0)\sin\omega_{dr}t_{i}+\frac{{\dot{q}}_{r}(0)+\xi_{r}\omega_{r}q_{r}(0)}{\omega_{dr}}\cos\omega_{dr}t_{i}\right) \end{array}$$

Displacement signals are intercepted and averaged by RDT. Then,

(9) $$\delta_{i}(s)=\frac{1}{n}\sum_{k=1}^{n}x_{i}(t_{i}+s)=\frac{1}{n}\sum_{k=1}^{n}\sum_{r=1}^{m}\varphi_{ir}q_{r}(t_{i}+s)=\sum_{r=1}^{m}\varphi_{ir}\left(\frac{1}{n}\sum_{k=1}^{n}q_{r}(t_{i}+s)\right)$$

This condition indicates that the problem of calculating the free vibration signal on the *i*th DOF ($\delta_{i}(s)$) can be transformed into the problem of segmented superposition and averaging of different orders of modal displacement responses through modal decomposition of linear structure.

(10) $$\begin{array}{l} \delta_{i}(s)=\sum_{r=1}^{m}\varphi_{ir}\left(\frac{1}{n}\sum_{k=1}^{n}q_{r}(t_{i}+s)\right)\\ =\sum_{r=1}^{m}\varphi_{ir}\left(\frac{1}{n}\sum_{k=1}^{n}\left(e^{-\xi_{r}\omega_{r}s}(A\cos\omega_{dr}s+B\sin\omega_{dr}s)\;\;+\int_{0}^{p}\varphi_{r}^{T}h_{r}(\tau )f(t_{i}+s-\tau )d\tau \right)\right)\\ =\frac{1}{n}e^{-\xi_{r}\omega_{r}s}\sum_{r=1}^{m}\varphi_{ir}\sum_{k=1}^{n}(A\cos\omega_{dr}s+B\sin\omega_{dr}s)\;\;+\frac{1}{n}\sum_{r=1}^{m}\varphi_{ir}\sum_{k=1}^{n}\left(\int_{0}^{p}\varphi_{r}^{T}h_{r}(\tau )f(t_{i}+s-\tau )d\tau \right)\\ =\frac{1}{n}e^{-\xi_{r}\omega_{r}s}\sum_{r=1}^{m}\varphi_{ir}\sum_{k=1}^{n}(A\cos\omega_{dr}s+B\sin\omega_{dr}s)\;\;+\sum_{r=1}^{m}\varphi_{ir}\int_{0}^{p}\varphi_{r}^{T}h_{r}(\tau )\left(\frac{1}{n}\sum_{k=1}^{n}\left(f(t_{i}+s-\tau )\right)\right)d\tau \end{array}$$

(11) $$\begin{array}{l} G=\frac{1}{n}e^{-\xi_{r}\omega_{r}s}\sum_{k=1}^{n}(A\cos\omega_{dr}s+B\sin\omega_{dr}s)\;\;\\ F=\frac{1}{n}\sum_{k=1}^{n}\left(f(t_{i}+s-\tau )\right) \end{array}$$

(12) $$\delta_{i}(s)=\sum_{r=1}^{m}\varphi_{ir}G+\sum_{r=1}^{m}\varphi_{ir}\int_{0}^{p}\varphi_{r}^{T}h_{r}(\tau )Fd\tau$$

Therefore, the signal gained by RDT under zero-mean non-stationary load can be divided into two parts. The first part is related only to the initial modal displacement and initial modal velocity of the structure, whereas the second part is related to non-stationary external excitation. Given that the mean of *F* is zero and the variance tends to approach zero with the increase in average times (*n*) of RDT, the second part of the signal gained by RDT approaches zero when *n* is large. The second part is viewed as noise. In other words, the signal gained by RDT under non-stationary excitation is a free vibration attenuation signal containing noises.

### 4.2. ARMA Model

The time series ARMA model is characterized by no energy leakage, strong noise resistance, and high identification accuracy in identifying the modal parameters of structures [28]. The relationship between N DOF linear system excitation and response can be described by the high-order differential equation

(13) $$[M]\left\{\ddot{X}\right\}+[C]\left\{\dot{X}\right\}+[K]\left\{X\right\}=\left\{F\right\}$$

where [M], [C], [K] are the mass, damping and stiffness matrix of the system respectively; {X}, $\left\{\dot{X}\right\}$, $\left\{\ddot{X}\right\}$, {F} are the displacement, velocity, acceleration response vector and excitation force vector of each point of the system.

In the discrete time domain, this high-order differential equation changes into a series of differential equations expressed by time series at different times. Therefore, the ARMA time series model equation is:

(14) $$\sum_{k=0}^{2N}a_{k}x_{t-k}=\sum_{k=0}^{2N}b_{k}f_{t-k}$$

where 2*N* is number of orders of the regression model and moving average model; $a_{k}$ and $b_{k}$ are the auto-regression coefficient and moving average coefficient for identification, respectively; and $f_{t}$ is the excitation of white noises. When *k* = 0, $a_{0}=b_{0}=1$.

Here $f_{t}$ is the white noise. Thus, the correlation function is:

(15) $$E[f_{t-i}f_{t+\tau -k}]=\left\{ \begin{array}{l} \sigma^{2}\;\;\;\;\;\;\;\;\;k=\tau +i\\ 0\;\;\;\;\;\;\;\;\;\;\;other \end{array}\right.$$

where $\sigma^{2}$ is the variance of white noise.

Given that the impulse response function ($h_{i}$) of the linear system is the output response of the system upon the excitation of impulse signal *δ*, the expression defined by an ARMA process is:

(16) $$\sum_{k=0}^{2N}a_{k}h_{t-k}=\sum_{k=0}^{2N}b_{k}\delta_{t-k}=b_{t}$$

Therefore,

(17) $$\sum_{k=0}^{2N}a_{k}R_{l-k}=\sum_{i=0}^{\infty }h_{i}\sum_{k=0}^{2N}a_{k}R_{i+l-k}=\sigma^{2}\sum_{i=0}^{\infty }h_{i}b_{i+l}$$

For an ARMA process, $b_{k}$ = 0 when *k* is larger than 2*N*. Hence, the above equation is equal to zero constantly when *l* > 2*N*. Then,

(18) $$\sum_{k=0}^{2N}a_{k}R_{l-k}=-R_{i}\;,\;\;\;\;\;\;\;\;\;l\;＞\;2N$$

Assuming that the length of the correlation function is *L* and let *M* = 2*N*. Corresponding to different *l* values, substituting them into the above equation yields a set of equations:

(19) $$\left\{ \begin{array}{l} a_{1}R_{M}+a_{2}R_{M-1}+\cdots +a_{M}R_{1}=R_{M+1}\\ a_{1}R_{M+1}+a_{2}R_{M}+\cdots +a_{M}R_{2}=R_{M+2}\\ \;\;\;\;\;\;\;\;\;\;\;\;\;\;\;\;\;\;\;\;\;\;\;\;\;\;\;\;\;\;\vdots \\ a_{1}R_{L-1}+a_{2}R_{L-2}+\cdots +a_{M}R_{L-M}=R_{L} \end{array}\right.$$

The least square solution of the equation set can be gained by the pseudo-inverse method:

(20) $$\left\{a\right\}={\left({\left[R\right]}^{T}\left[R\right]\right)}^{-1}\left({\left[R\right]}^{T}\left[R^{\prime }\right]\right)$$

Therefore, the auto-regression coefficient $a_{k}(k=1,2,\cdots ,2N)$ could be calculated.

The coefficient of the moving average model ($b_{k}(k=1,2,\cdots ,2N)$) could be solved by using the following nonlinear equation set:

(21) $$\left\{ \begin{array}{l} b_{0}^{2}+b_{1}^{2}+\cdots b_{M}^{2}=c_{0}\\ b_{0}b_{1}+\cdots +b_{M-1}b_{M}=c_{1}\\ \;\;\;\;\;\;\;\;\;\;\;\;\;\;\;\;\;\vdots \\ b_{0}b_{M}=c_{M}\\ c_{k}=\sum_{i=0}^{2N}\sum_{j=0}^{2N}a_{i}a_{j}C_{k-i+j}\;\;,\;\;\;\;\;\;\;\;k=0,1,2,\cdots ,2N \end{array}\right.$$

where $C_{k}$ is the auto-covariance function of the response sequence *x_t_*.

After $a_{k}$ and $b_{k}$ are gained, the modal parameters of the system can be calculated by the transmission function of the ARMA model. The transmission function of the ARMA model is:

(22) $$H(z)=\frac{\sum_{k=0}^{2N}b_{k}z^{-k}}{\sum_{k=0}^{2N}a_{k}z^{-k}}$$

Roots of the denominator polynomial equation can be calculated by the high-order algebraic equation:

(23) $$z^{2N}+a_{1}z^{2N-1}+\cdots +a_{2N-1}z+a_{2N}=0$$

The calculated roots are polar points of the transmission function. Their relationships with modal frequency ($\omega_{k}$) and damping ratio ($\xi_{k}$) of the system are:

(24) $$\left\{ \begin{array}{l} z_{k}=\exp(s_{k}\Delta t)=\exp[(-\xi_{k}\omega_{k}+j\omega_{k}\sqrt{1-\xi_{k}{}^{2}})\Delta t]\\ z_{k}{}^{*}=\exp(s_{k}{}^{*}\Delta t)=\exp[(-\xi_{k}\omega_{k}-j\omega_{k}\sqrt{1-\xi_{k}{}^{2}})\Delta t] \end{array}\right.$$

Based on Equation (24), $\omega_{k}$ and $\xi_{k}$ can be calculated as follows:

(25) {Rk=lnzk=skΔtkωr=|Rk|/Δtξk=11+(Im(Rk)/Re(Rk))2

## 5. Engineering Applications and Discussions

### 5.1. Stability Test of Equipment

To ensure the accuracy and stability of the testing equipment, the stability of all equipment was tested before the experiment. In the experiment, the Haixingda H32 receiver of Zhonghaida Company was used as the GNSS-RTK receiver, and the sampling frequency was adjusted from 1 Hz to 20 Hz through internal upgrading. The positioning accuracy of RTK was ±(10 mm + 1 ppm) on plane and ±(20 mm + 1 ppm) on elevation.

**Figure 4:**
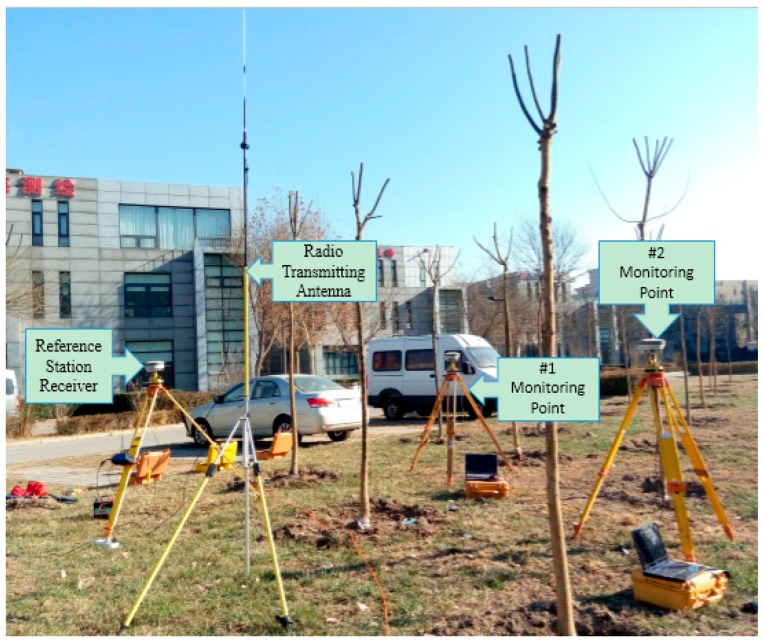
Stability test site.

**Figure 5:**
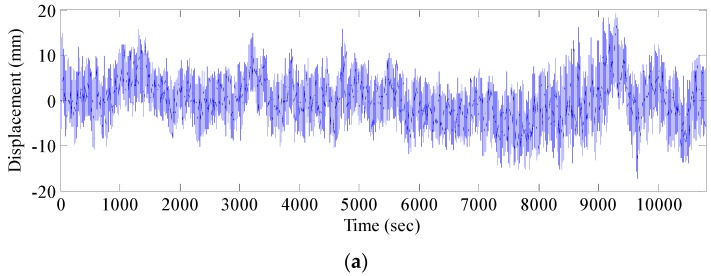

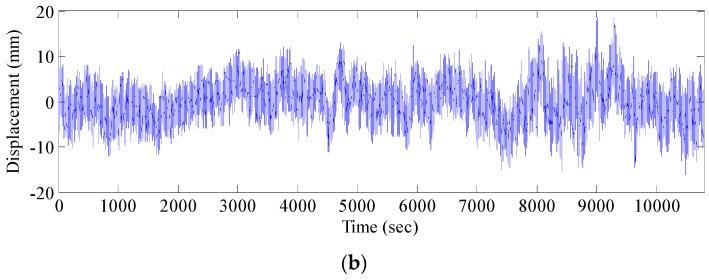
Stability test data: (**a**) #1 monitoring point elevation data; and (**b**) #2 monitoring point elevation data.

The experiment was conducted with three GPS receivers on an open site. One was used as the reference station, while the remaining two were used as mobile stations for mutual verification (Figure 4). Data were collected for three continuous hours. Elevation data in 3 h were used, and the original data are shown in Figure 5. Figure 5 shows that the vertical accuracy of two pieces of equipment is within 20 mm, indicating the accuracy reliability and stability of the test equipment.

### 5.2. Engineering Background and Measuring Point Arrangement

**Figure 6:**
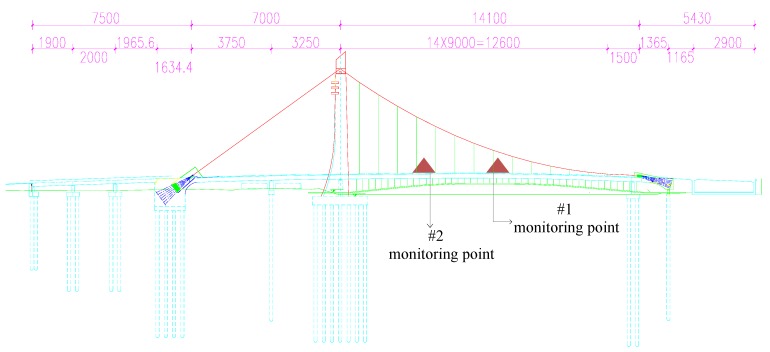
The elevation view of Fumin Bridge and the test point arrangement.

The Tianjin Fumin Bridge is in a central urban area. The main bridge is a self-anchored suspension bridge with a single tower and spatial cables. The tower is supported by one column. The main span cable is anchored at two sides of the girder, and the side span cable is anchored onto the ground, forming a stable structural system. The main span cable uses a 3D spatial line, which is a parabola on the vertical and horizontal surfaces. The side span cables use a group (two parallel lines) of cables without vertical sling (Figure 6). The total length of the bridge is 340.6 m, including 157.081 m main span and 86.4 m side span. The approach bridge at the east of the river is (19 + 20 + 19.6) m (three-span ordinary reinforced concrete continuous beams), and the approach bridge at the west of the river (single-span concrete framework with cantilevers) is 38.219 m.

The standard width of the main bridge is 38.6 m. The transversal arrangement is 0.8 m (cable anchorage region) + 0.5 m (crash barrier) + 14.5 m (3.75 m non-motorized vehicle lane + (3.75 + 2 × 3.5) m motorway) + 0.5 m (marginal strip) + 0.5 m (crash barrier) + 5.0 m (main tower region) +0.5 m (crash barrier) + 0.5 m (marginal strip) + 14.5 m ((2 × 3.5 + 3.75 m) non-motorized vehicle lane + 3.75 m motorway) + 0.5 m (crash barrier) + 0.8 m (cable anchorage region).

**Figure 7:**
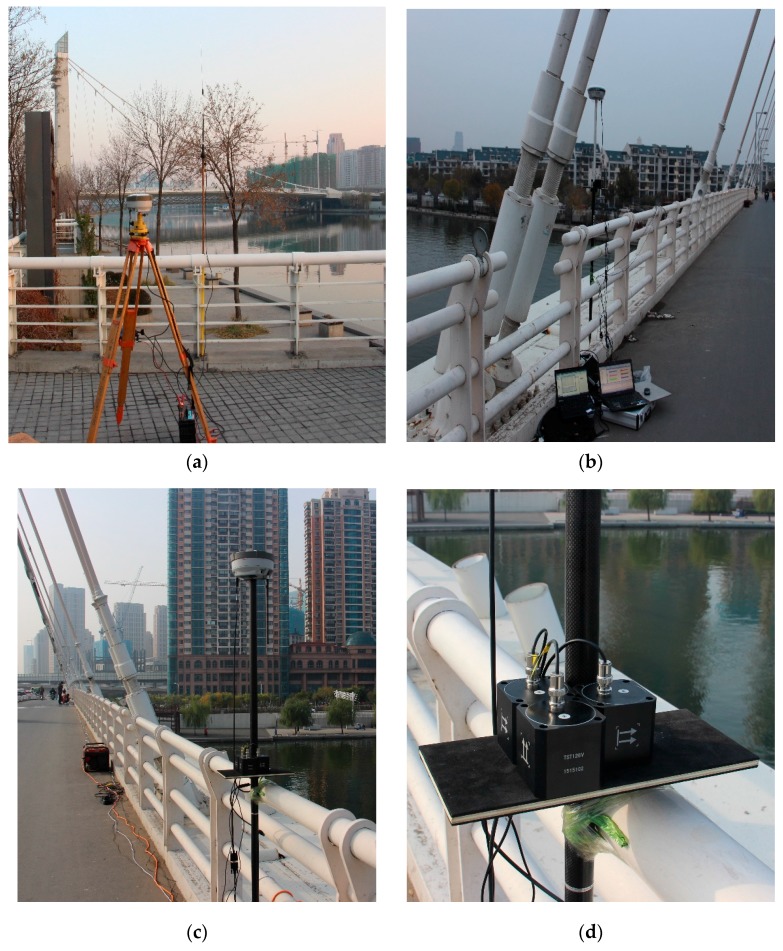
Instruments layout where (**a**) is the reference station; (**b**) is the #1 mobile station; (**c**) is the #2 mobile station and (**d**) is the arrangement of accelerometers.

In consideration of the characteristics of the Fumin bridge structure and experimental goal, only the main-span bridge was monitored. Two monitoring points were set at 1/4 and 1/2 of the main-span bridge. One GNSS-RTK receiver and one acceleration sensor were set at each monitoring point. A GNSS-RTK receiver–acceleration sensor combined monitoring device was designed. The reference station is shown in Figure 7a. The #1 and #2 moving stations are shown in Figure 7b,c. The installation acceleration sensor is shown in Figure 7d. The longitudinal, horizontal, and vertical acceleration data of the bridge structure were tested.

### 5.3. Identification of Dynamic Displacement from AFEC Mixed Filtering

**Figure 8:**
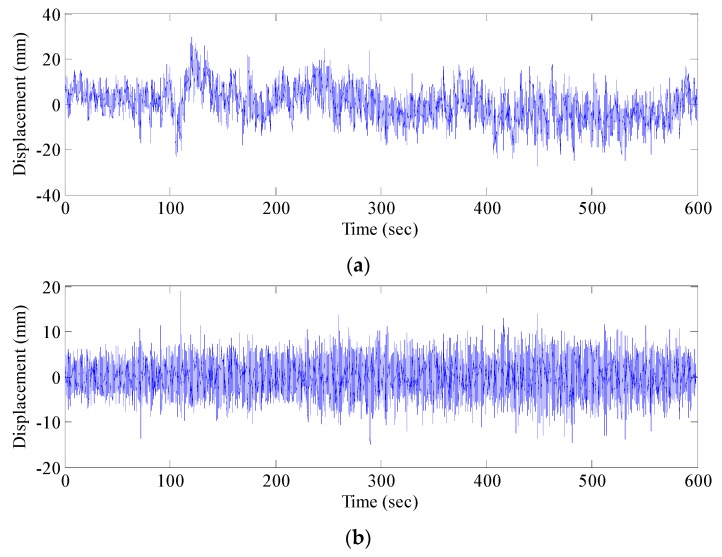
GNSS-RTK elevation signal: (**a**) original signal-$x_{1}$; and (**b**) signal after pretreatment-*X*_1_.

Dynamic responses of the Fumin Bridge under ambient excitations were monitored for 10 successive hours from 10:00 a.m. to 8:00 p.m. on 9 November 2016. The GNSS sensor collected the vibration displacement signal of the structure, while the accelerometer collected the vibration acceleration signal of the structure. Four groups of data were collected. The effective number of satellite signals that has been used in the measurements is 14–16, and it was constant throughout the test. The GNSS sensor and accelerometer recorded original data at the sampling frequencies of 20 Hz and 100 Hz, respectively. The calculated results of RTK were transmitted and stored in laptops through USB cables. The original data $x_{i}$ were preprocessed by deleting abnormal values according to the principle of triple standard deviation (99.7% confidence interval) and repairing missed data by cubic spline interpolation and data smoothing by the moving average method, thus obtaining the signal after preprocessing (*X_i_*). The original vertical displacement sequence (*x*_1_) and signal after preprocessing (*X*_1_) at the #1 measuring point are shown in Figure 8.

**Figure 9:**
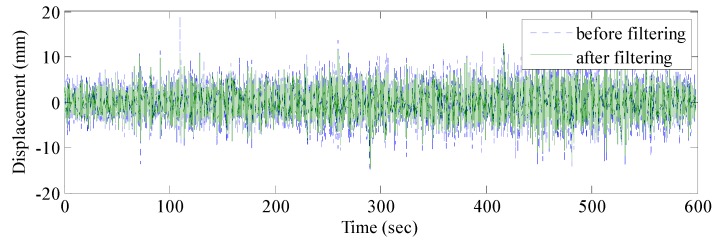
GNSS-RTK signal before and after Chebyshev filtering.

**Figure 10:**
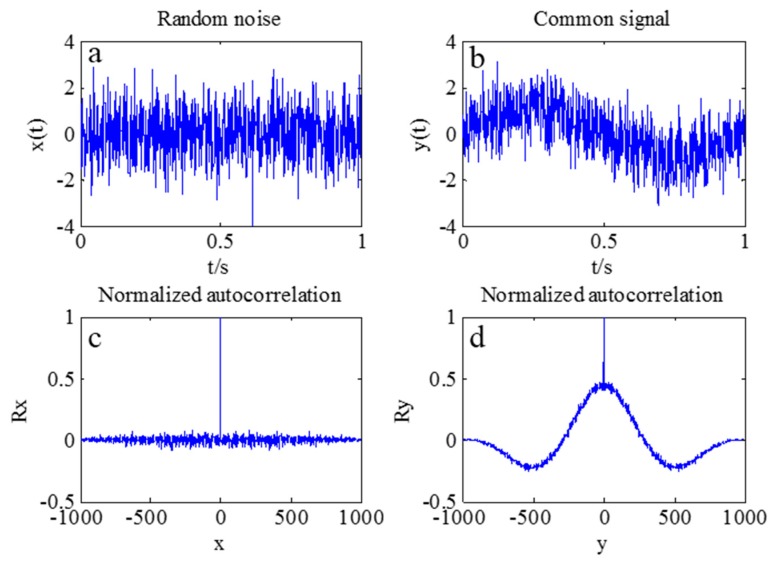
Normalized autocorrelation of the random noise and common signal. (**a**) Random noise; (**b**) Common signal; (**d**) Normalized autocorrelation and (**d**) Normalized autocorrelation.

The real vibration data of the structure in the GNSS data were covered by noises due to the interferences caused by multipath errors and random noises. According to Section 3, the AFEC mixed filtering algorithm was applied to process the polluted GNSS data. First, an 8-order I-type Chebyshev high-pass filter was used to eliminate multipath errors (Figure 9). Second, the random noise error was reduced by the EMD based on autocorrelation function. The autocorrelation function of the signal reflects correlations at different times because of the statistical characteristics of the random signal. The autocorrelation function of random noise is characterized by a maximum at zero, a rapid decay to very little at other points. There is no such rule for the ordinary signal because the ordinary signal has a correlation between different moments. It is possible to distinguish whether the IMF component is a noise component by determining whether the autocorrelation function characteristic of the IMF component complies with the autocorrelation function characteristic of the random noise signal. Therefore, the random noise and ordinary signal can be distinguished by the autocorrelation function of signals. The autocorrelation function between the random noises and ordinary signal is presented in Figure 10.

**Figure 11:**
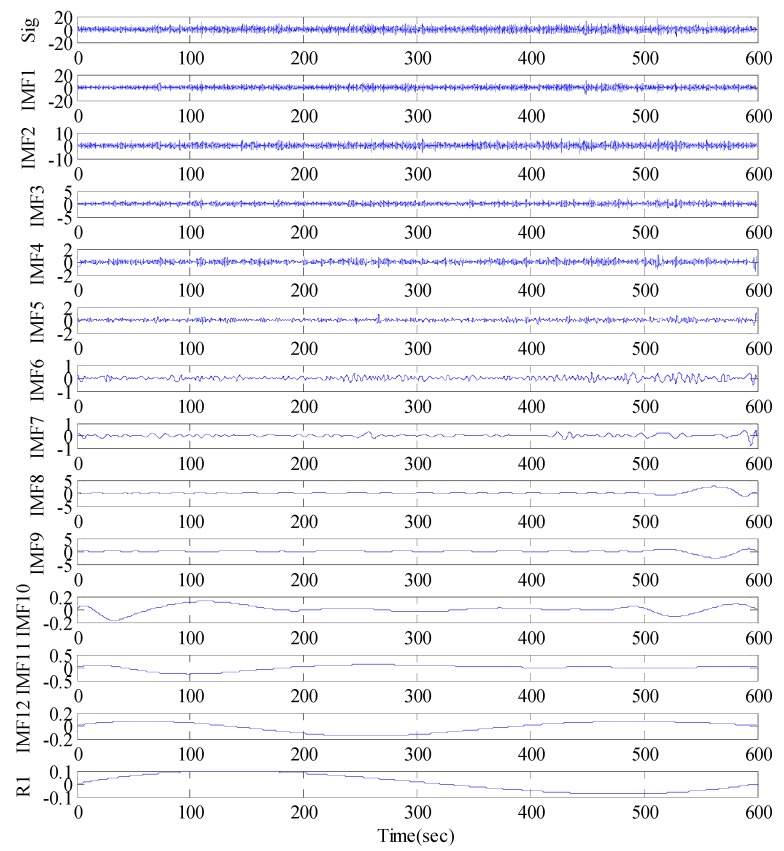
EMD (Empirical Mode Decomposition) decomposition of GNSS-RTK signal.

**Figure 12:**
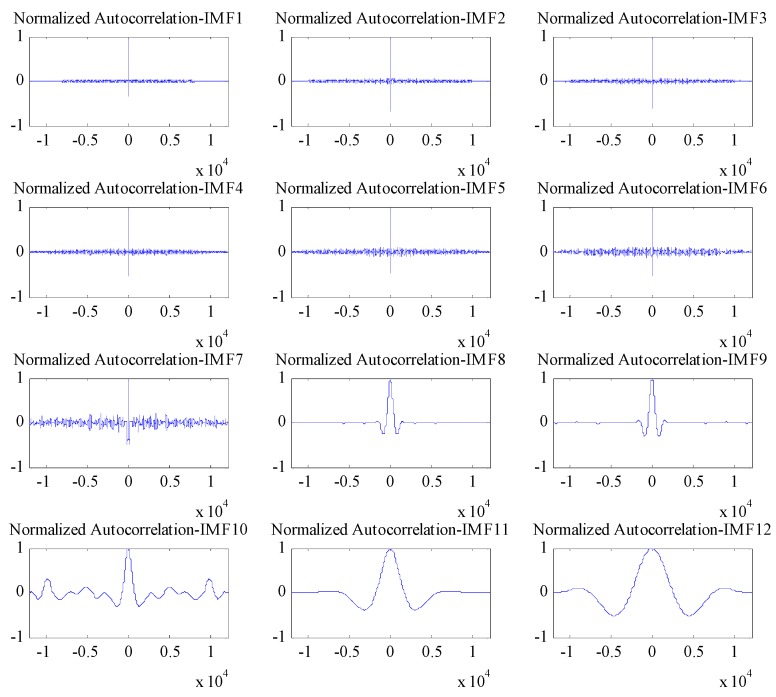
Normalized autocorrelation of IMFs (Intrinsic Mode Functions).

**Figure 13:**
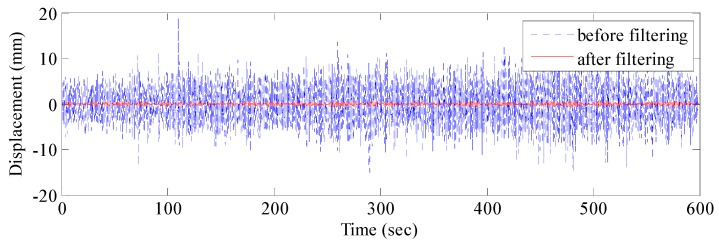
Comparison of elevation signal before and after AFEC filtering.

**Figure 14:**
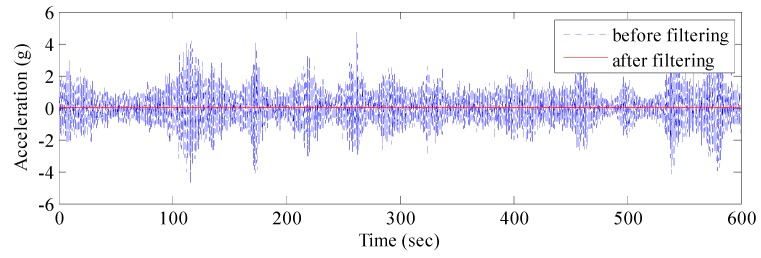
Comparison of acceleration signal before and after AFEC filtering.

The EMD of the signal was conducted first (Figure 11), which provided 12 IMF components and one residual component. Subsequently, the normalized autocorrelation functions of 12 IMFs were solved (Figure 12). In Figure 12, the first seven IMFs conformed to the characteristics of random noise. The AFEC-filtered signal were reconstructed with the five remaining IMFs and R1. The real vibration amplitude of the bridge structure that was extracted by the AFEC de-noising was less than 1 mm, showing high accuracy. The comparison between the real vibration signals before and after AFEC de-noising is shown in Figure 13. Given that the vibration frequency of the bridge structure generally ranges between 0.1–10 Hz, only the 0–10 Hz frequency spectrum was analyzed by power spectral analysis. Acceleration signals within 0–10 Hz were collected using the Chebyshev band-pass filter method. Signals before and after filtering were compared (Figure 14).

**Figure 15:**
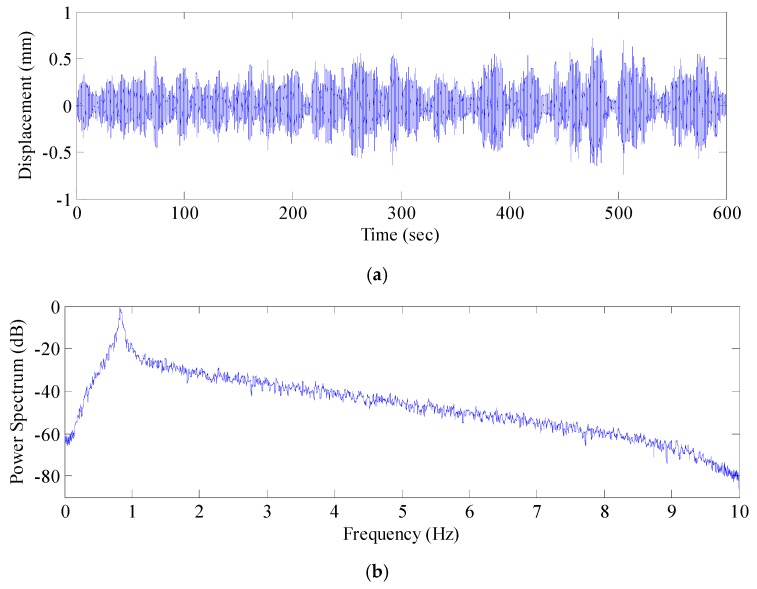
GNSS-RTK signal after AFEC (an combination algorithm of autocorrelation function-based empirical mode decomposition (EMD) and Chebyshev mixed filtering) and power spectrum analysis: (**a**) GNSS-RTK signal after AFEC; (**b**) Power spectrum analysis of GNSS-RTK signal after AFEC.

**Figure 16:**
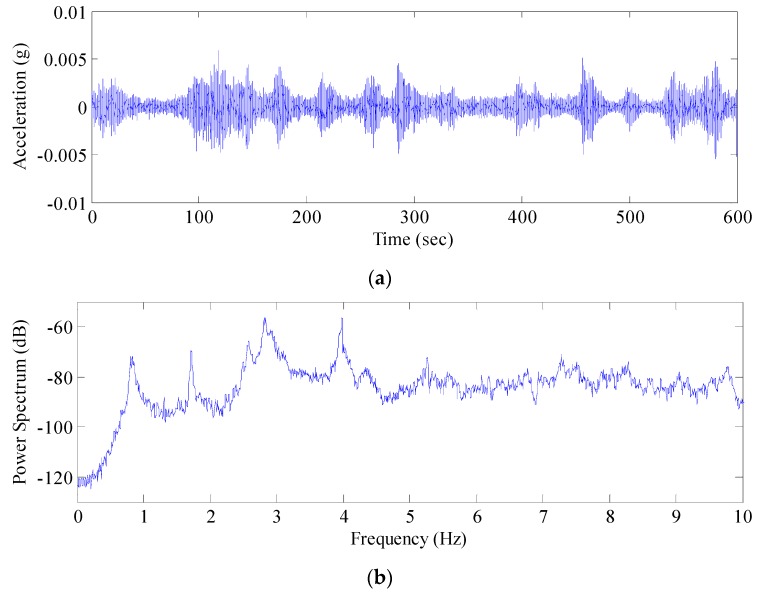
Acceleration signal after AFEC and power spectrum analysis: (**a**) acceleration signal after AFEC; (**b**) Power spectrum analysis of acceleration signal after AFEC.

**Figure 17:**
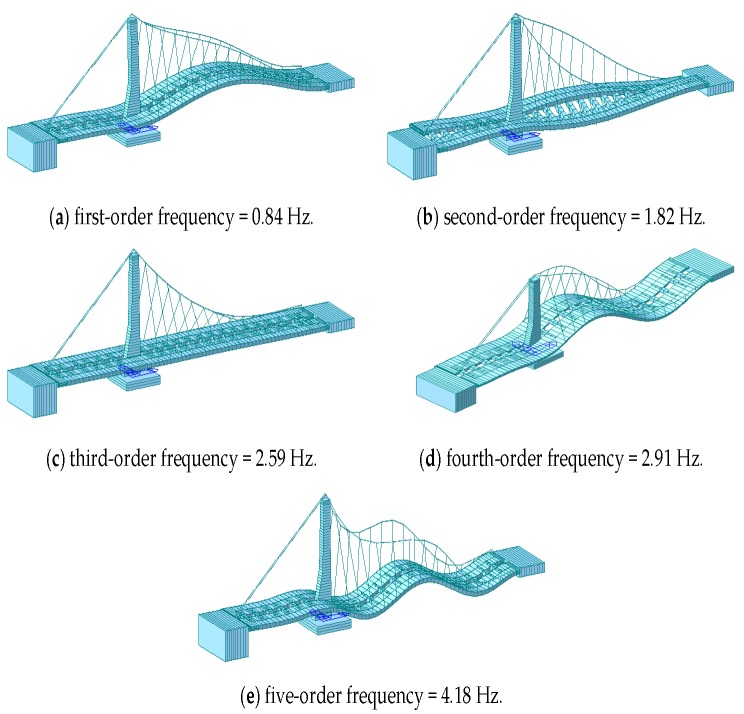
Finite element modal analysis: (**a**) First order mode; (**b**) second-order mode; (**c**) third-order mode; (**d**) fourth-order mode; (**e**) five-order mode.

Next, the power spectra of the de-noised displacement sequence and the acceleration signal sequence were analyzed (Figure 15 and Figure 16). The AFEC-filtered GNSS-RTK displacement sequence identified the frequency of the bridge structure (0.82 Hz), and the filtered acceleration signal sequence identified the first five orders of frequency of the bridge structure, valued at 0.84 Hz, 1.82 Hz, 2.59 Hz, 2.91 Hz, and 4.18 Hz, respectively. The identification results were close to the finite element analysis results (Figure 17). The MIDAS Civil 2012 was used to establish the 3-dimensional finite element theoretic calculation model for the Tianjin Fumin Bridge to calculate the self-vibration modal parameters of the structure. In the calculation and analysis model, cable and suspender are simulated by cable element. Moreover, girder and tower are simulated by beam element. The model contains 625 nodes, 62 cable units and 882 beam elements.

### 5.4. Modal Analysis

**Figure 18:**
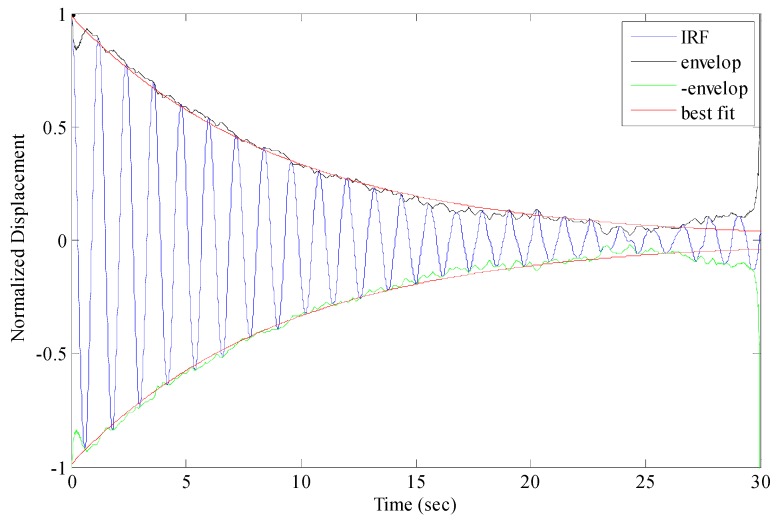
Free-damped vibration signal obtained from RDT.

The GNSS-RTK signals filtered by the AFEC mixed filtering algorithm were analyzed using the expanded ARMA_RDT modal identification method. First, the free attenuation signals of the bridge structure were collected by the expanded RDT (Figure 18). Second, the modal parameters were identified using the free attenuation signals as the input signal of the ARMA method. The frequency identification results are listed in Table 1. Table 1 shows that the expanded ARMA_RDT modal identification method can effectively extract the first five orders of frequencies of the bridge structure in the GNSS-RTK data and that the maximum relative error is 5.4%, showing high identification accuracy.

## 6. Conclusions

The real-time dynamic responses of the Tianjin Fumin Bridge were monitored by combining the GNSS-RTK dynamic displacement measurement technique and the acceleration sensor system. Multipath errors and random noise in the GNSS monitoring data were eliminated by the AFEC mixed filtering algorithm, thus extracting real dynamic displacement sequences of the bridge structure. Next, the modal parameters of the bridge structure during operation were identified by the expanded ARMA_RTD modal identification method, which identified the first five orders of the natural frequency of vibration. The identification results were close to the test results of the accelerator sensor and the theoretical calculation results of the finite element analysis. The maximum error of the natural frequency of vibration was within 6%. Some conclusions were reached in this paper:

(1) The GNSS technique possesses a certain engineering application value in vibration displacement monitoring and modal parameter identification of large engineering structures.
(2) The proposed AFEC mixed filtering algorithm can not only eliminate multipath errors and random noise in the GNSS-RTK data effectively but can also increase the vertical vibration displacement accuracy of the bridge structure to less than 1 mm.
(3) The expanded ARMA_RTD modal identification method can be used to process the GNSS monitoring data of real bridge structures and can identify the modal parameters of the structure quickly and effectively. It can provide key data for online monitoring or active control and the earthquake resistance evaluation of structures.

With the further development of the GNSS technique, sampling frequency increases. On the basis of data testing and analysis, the GNSS-based dynamic deformation monitoring technique is not only flexible and easy to operate but can also be feasibly used in the dynamic weighing and damage identification of bridge structures. It possesses promising application prospects.

## Figures and Tables

**Table 1:** Comparison study of the first five frequency data sets.

Frequency (Hz)	First-Order	Second-Order	Third-Order	Fourth-Order	Five-Order
Theoretical value	0.84	1.82	2.59	2.91	4.18
Result from Accelerometer	0.82	1.71	2.57	2.82	3.98
Relative difference (%)	2.38	6.04	0.77	3.09	4.78
Result from GNSS-RTK	0.81	1.77	2.45	2.78	4.03
Relative difference (%)	3.57	2.75	5.4	4.47	3.59
